# Genome of a Low-Salinity Ammonia-Oxidizing Archaeon Determined by Single-Cell and Metagenomic Analysis

**DOI:** 10.1371/journal.pone.0016626

**Published:** 2011-02-22

**Authors:** Paul C. Blainey, Annika C. Mosier, Anastasia Potanina, Christopher A. Francis, Stephen R. Quake

**Affiliations:** 1 Howard Hughes Medical Institute, Department of Bioengineering, Stanford University, Stanford, California, United States of America; 2 Department of Environmental Earth System Science, Stanford University, Stanford, California, United States of America; Argonne National Laboratory, United States of America

## Abstract

Ammonia-oxidizing archaea (AOA) are thought to be among the most abundant microorganisms on Earth and may significantly impact the global nitrogen and carbon cycles. We sequenced the genome of AOA in an enrichment culture from low-salinity sediments in San Francisco Bay using single-cell and metagenomic genome sequence data. Five single cells were isolated inside an integrated microfluidic device using laser tweezers, the cells' genomic DNA was amplified by multiple displacement amplification (MDA) in 50 nL volumes and then sequenced by high-throughput DNA pyrosequencing. This microscopy-based approach to single-cell genomics minimizes contamination and allows correlation of high-resolution cell images with genomic sequences. Statistical properties of coverage across the five single cells, in combination with the contrasting properties of the metagenomic dataset allowed the assembly of a high-quality draft genome. The genome of this AOA, which we designate Candidatus *Nitrosoarchaeum limnia* SFB1, is ∼1.77 Mb with >2100 genes and a G+C content of 32%. Across the entire genome, the average nucleotide identity to *Nitrosopumilus maritimus*, the only AOA in pure culture, is ∼70%, suggesting this AOA represents a new genus of Crenarchaeota. Phylogenetically, the 16S rRNA and ammonia monooxygenase subunit A (*amoA*) genes of this AOA are most closely related to sequences reported from a wide variety of freshwater ecosystems. Like *N. maritimus*, the low-salinity AOA genome appears to have an ammonia oxidation pathway distinct from ammonia oxidizing bacteria (AOB). In contrast to other described AOA, these low-salinity AOA appear to be motile, based on the presence of numerous motility- and chemotaxis-associated genes in the genome. This genome data will be used to inform targeted physiological and metabolic studies of this novel group of AOA, which may ultimately advance our understanding of AOA metabolism and their impacts on the global carbon and nitrogen cycles.

## Introduction

Mesophilic Crenarchaea (first discovered by Fuhrman et al. [1] and DeLong [2]) are among the most abundant microorganisms on the planet [1]–[2]. Crenarchaea constitute ∼10–40% of the total microbial community in the ocean below the euphotic zone [3]–[6]. In fact, it is estimated that there are 1.0×10^28^ crenarchaeal cells in the world's oceans compared to 3.1×10^28^ bacterial cells [3]. In soil systems, Crenarchaea generally comprise 1–5% of the total prokaryotic community [7]–[9], but can reach upwards to 38% in some systems (*e.g.*, temperate acidic forest soils) [10]. It appears that the vast majority of Crenarchaea in soils and the open ocean possess ammonia monooxygenase (*amoA*) genes and thus may be capable of oxidizing ammonia to nitrite, based on quantitative PCR estimates of 16S rRNA and *amoA* genes, fluorescent *in situ* hybridization, and metagenomic analyses [11]–[18]. Recent phylogenetic and genomic analyses suggest that these mesophilic Crenarchaea should be recognized as a third archaeal phylum, the Thaumarchaeota [19]–[20].

AOA have proven difficult to isolate in culture, as evidenced by the fact that only one pure culture [21] has been documented despite reports of several enrichment cultures [12], [22]–[25]. Many questions remain about the physiology, metabolic and biogeochemical functions, evolutionary history, and ecological niche partitioning of AOA. Genome sequencing is a powerful tool that can be used to address these outstanding questions, yet traditional sequencing approaches require pure cultures or enrichment to near homogeneity. Genome sequencing of individual cells presents a new approach for exploring the genetic makeup of microorganisms present in heterogeneous mixtures (such as the enrichment culture that was the basis for this study) without the need for cell viability or growth. Single-cell genomics can be used independently on uncultured microbes [26]–[27] or as a complement to metagenomic studies on enrichment cultures and environmental samples by aiding in sequence binning and genome reconstruction [28].

Here, we used a combination of single-cell genomic and metagenomic approaches to sequence the genome of a novel, low salinity-type AOA. The single-cell approach is useful for obtaining organism-specific sequence datasets from heterogeneous samples, and furthermore provides an insight into micro-heterogeneity at the population level. The genomic data reveal sequence features not observed in other AOA, as well as similarities to published AOA genomes, which speak to the metabolic potential and environmental relevance of these organisms.

## Results and Discussion

### Enrichment of ammonia-oxidizing archaea from low-salinity sediments

**Figure 1:**
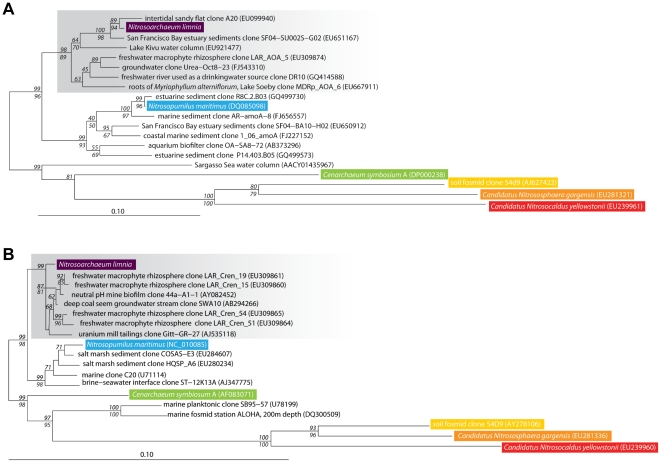
Phylogeny of Ammonia Oxidizing Archaea gene sequences. Neighbor-joining phylogenetic tree of (A) archaeal *amoA* gene sequences and (B) archaeal 16S rRNA gene sequences. Grey boxes highlight the putative low-salinity group. Significant bootstrap values (≥50) from 1000 replicates are shown in italics at branch nodes. Neighbor-joining bootstrap values are above the line and parsimony values are below the line.

An ammonia-oxidizing enrichment culture initiated from sediments in the low-salinity region of San Francisco Bay was dominated by AOA; CARD-FISH showed that Archaea (Arch915 probe) accounted for approximately 84% of cells in the enrichment and the remaining 16% were Bacteria (Eub338 I–III probes). The AOA grew chemoautotrophically by aerobic ammonia oxidation to nitrite. Archaeal *amoA* and 16S rRNA gene sequences from the AOA enrichment culture were phylogenetically distinct (bootstrap supported) from *N. martimus* [Figure 1], and were instead most closely related to environmental clones derived from freshwater ecosystems. In total, over 500 GenBank *amoA* sequences fell within this phylogenetic cluster, 79% of which were from low salinity habitats. Another 7% of the sequences were from habitats that experience low salinity at some point in the year (e.g., San Pablo Bay sites in San Francisco Bay), and 4% of the sequences were from soil. Most of the remaining sequences were from salt marsh sediments [29], suggesting that this AOA phylotype may tolerate elevated salinity. Nevertheless, the vast majority of *amoA* sequences within this clade were from freshwater habitats.

On the basis of these results and the genome analysis described herein, we propose the provisional classification of this novel archaeon as Candidatus *Nitrosoarchaeum limnia* SFB1. The name refers to low-salinity habitat of this archaeon and to its ability to oxidize ammonia to nitrite. *Nitrosoarchaeum* comes from the Latin masculine adjective ‘nitrosus’ for nitrous and *limnia* is derived from the Greek word ‘limne’ for freshwater (as in limnology) and the strain name SFB1 refers to its isolation from San Francisco Bay. The short description of Candidatus *Nitrosoarchaeum limnia* is a mesophilic, chemolithoautotrophic ammonia-oxidizing archaeon belonging to the Thaumarchaeota phylum.

### On-chip cell sorting

Based on this information suggesting that *N. limnia* is novel and distinct from *N. maritimus*, we employed a combined single cell sorting and metagenomic approach to obtain the genome sequence of these AOA. A variety of single-cell approaches to microbial genomics are currently being applied [26]–[27], [30]–[31]. While all rely on the multiple displacement amplification (MDA) reaction [32] for the amplification of unknown genomic DNA sequences, the means by which individual cells are isolated distinguish the various approaches. Microfluidic flow [30], flow cytometry [31], [33], and micromanipulation [27] have all been successfully applied to isolate single cells for genomic amplification, although each approach has strengths and weaknesses. In particular, microfluidics and micromanipulation allow microscopic examination of the sorted cells, while flow cytometry provides higher raw throughput. A challenge in this case was the tiny, submicron size of the AOA in the enrichment culture.

We implemented laser tweezer [34]–[35] based cell sorting inside a microfluidic device to combine the benefits of a sealed, integrated system for sorting and amplification, small reaction volumes, and minimum sorting volume. The imaging and trapping system was adapted to enable visualization and trapping of tiny cells. This required the application of a high-numerical aperture phase-contrast objective with high infrared transmission. All reagents were carefully filtered to remove extraneous particles that could be confused with cells. These features of the optical sorting method specifically and effectively address the three classes of contamination with the potential to compromise single cell amplification reactions, namely: the laboratory environment, amplification reagents, and cells/DNA from the sample itself.

Using this microfludic and laser trapping device, single cells smaller than one micron in length were sorted from the AOA enrichment culture [Figure S1]. The cells were lysed ‘on-chip’ using proteinase K and alkaline lysis, and the genomes of each individual cell were amplified by MDA. Amplified cells were screened for archaeal *amoA* and 16S rRNA genes to confirm affiliation with AOA. Based on PCR screening, we selected the MDA products of five individual AOA cells for genomic sequencing. In addition, due to the highly enriched nature of the ammonia-oxidizing culture (with respect to the AOA), we also carried out MDA on a benchtop scale using cells from the enrichment culture as the template. We directly sequenced the enrichment MDA products to obtain sequences from the ‘bulk’ enrichment culture metagenome. This combined single cell/metagenomic approach enabled us to test the single cell assemblies for completeness and the metagenome for contamination.

### Sequencing and assembly

MDA-amplified genomic DNA from the five individual AOA cells was sequenced using a combination of shotgun (SG) and paired-end (PE) DNA pyrosequencing approaches on the Roche/454 FLX Titanium platform. MDA products from both individual cells and the bulk enrichment were used to create sequencing libraries, which were quantitated using digital PCR [36] and sequenced. We analyzed 483,350 SG and 56,122 PE reads (119 Mb) obtained from five individual cells and 160,632 SG and 18,118 PE reads (37 Mb) from the enrichment sample [Table 1]. The G+C contents of the single-cell reads formed a major peak centered at 32.4%, an even lower G+C content than *N. maritimus* (34.2%) [Figure S2]. To compare the dispersion of the G+C content, we simulated shotgun reads from *N. maritimus* with the same average read length as the experimentally derived datasets. The dispersion of the read G+C content from the single-cell reads was essentially identical to that found of *N. maritimus*, confirming that the individual AOA cells were amplified without contamination from bacterial cells or DNA. The G+C content of the enrichment reads demonstrated a principal mode similar to the single cell datasets and *N. maritimus* (suggesting a large proportion of AOA in the enrichment), as well as a higher G+C component attributed to sequences from the bacteria present in the culture.

**Table 1:** *N. limnia* assembly statistics for the consensus genome (metagenome and five single cells), single cell coassembly (five single cells), and three individual cell assemblies.

assembly statistic	Consensus	Single cells only	Cell 90507.23	Cell 90507.21	Cell 90507.3
raw reads	693,589	530,748	70,156	200,938	138,886
paired-end reads	33,725	26,953	3,018	517	2,388
raw bases	150,994,537	118,796,782	17,107,411	52,341,561	29,999,202
assembly bases	1,769,573	1,690,404	1,094,113	1,039,820	1,041,604
number of elements	31	136	355	253	348
number of scaffolds	2	26	68	76	83
scaffold bases	1,757,035	1,366,809	852,837	892,230	834,370
scaffold N50	1,363,261	155,965	20,543	16,443	14,147
largest scaffold	1,363,261	263,850	70,423	49,773	41,297
contig bases	1,740,160	1,664,128	1,090,610	1,039,764	1,031,668
contig N50	94,192	28,182	14,827	14,834	9,613
unscaffolded contigs	29	110	287	177	265
largest contig	230,803	135,296	70,423	49,773	27,947

Individually, three of the single-cell datasets gave assemblies in excess of one megabase from sequencing depths of 10× to 30×, based on an expected genome size of about 1.7 Mb. The implied 60% genomic coverage is typical in our experience with single-cell genomic data at such depths of sequencing. The limited genomic coverage of the single-cell assemblies owes to the phenomenon of MDA amplification bias, wherein certain regions of the genome are amplified to a greater extent than other regions. The less-amplified regions are not efficiently sampled by the shotgun sequencing procedure, and extremely high sequence coverage is required to recover the under-represented sequences.

Interestingly, each of these single-cell assemblies represents a different 60% of the target genome, indicating that the genomic coverage and/or amplification bias profile differs from cell to cell. This phenomenon is consequential for single-cell genomic analyses, and can be utilized to improve study outcomes when considered *vis a vis* project goals at the stages of cell screening, DNA sequencing, assembly and analysis. Specifically, the lack of correlation among the amplification profiles of independently amplified cells allows the improvement of coverage profiles by combining datasets from different individual cells. Thus, while further sequencing and efforts to improve the assembly suffer diminishing returns with respect to an individual cell, great strides in assembly quality are quickly won by combining the reads obtained from several individual cells. By pooling the data from the five individual cells sequenced, we obtain an assembly of 136 elements (26 scaffolds and 110 contigs) for a total of 1.69 Mb of sequence, 1.37 Mb of which was captured in the scaffolds (scaffold N50 = 156 kb) [Table 1]. A combination of rarefaction analysis [Figure 1] and comparison to the metagenomic data indicates (Table 1) that this single-cell assembly represents >95% of the *N. limnia* genome.

The metagenome from the bulk enrichment was used as an independent validation of the single cell data and to supplement the single cell analysis. To combine the strengths of each dataset—the pure single cell AOA reads with no bacterial sequences and the bulk enrichment reads with less amplification bias—we generated a high-quality consensus assembly. First, we used five single-cell datasets to establish a stringent G+C content filter and eliminate high G+C content (>46%) reads from the enrichment dataset, purging much of the bacterial sequence contribution prior to assembly of the data. Next, we mapped the single-cell reads to a *de novo* assembly of the filtered enrichment reads to identify and reject chimeric sequences, the occurrence of which is expected to be independent in different datasets. The small pool of high-G+C reads was mapped against *N. maritimus* and *C. symbiosum* A to recover high G+C content conserved sequences. The consensus assembly contained 2 scaffolds and 29 contigs for a total of 1.77 Mb (contig N50 = 94 kb) [Table 1]. More than 99% of the assembled bases were represented by the two scaffolds (1.36 Mb and 0.39 Mb in length). The consensus assembly represents greater than 97% of the genome, based on rarefaction analysis. The elements from both assemblies were oriented and ordered by homology to *N. maritimus*. Orientation was determined by maximizing the sum of the alignment score to this reference, and the elements ordered according to the hit length-weighted average subject position of the homologous regions that were co-orientated with the reference. The genome sequence is reported in the supplementary information file Data S1.

### Microheterogeneity and sequencing (MDA) error

Comparing homologous regions of the single cell assemblies with each other and with the enrichment dataset, we consistently find 99.9% average nucleotide identity. The identity result is a lower limit because of the possibility of errors introduced in the amplification and sequencing procedures. Newbler reports 5904 bases in the assembly as having quality scores lower than 40. If we conservatively estimate the average quality score of these lower-quality aligned bases at 10 and the average quality score of the high-quality aligned bases at 40, 234 errors arising from ambiguous alignments and low-quality raw bases are predicted. Based on independent measurements (data not shown), we expect a minor contribution of additional errors based on the MDA procedure, on the order of 1/100,000 bp, or fewer than 20 bases across the *N. limnia* genome. Thus, it seems that microheterogeneity among the enrichment AOA must account for a substantial portion of the SNPs predicted among the individual cells.

### Genome size estimate

**Figure 2:**
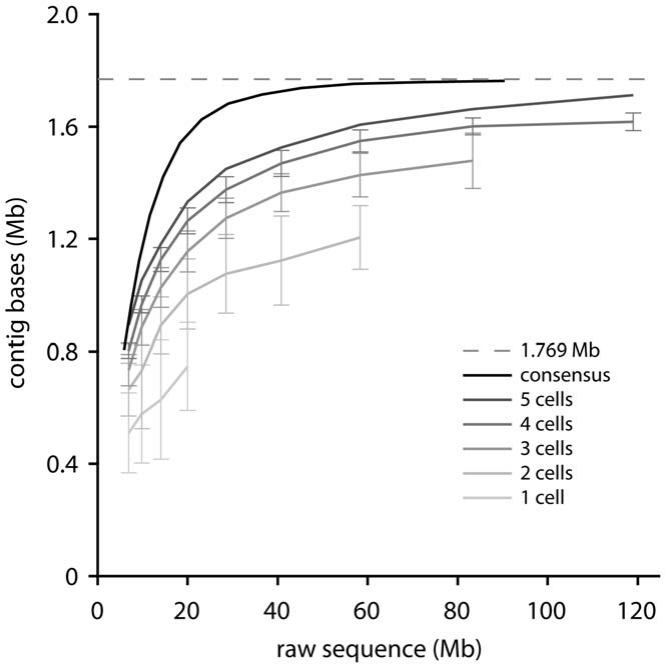
Assembly of the N. limnia datasets. Rarefaction analysis of the *N. limnia* sequence data showing assembly of the consensus dataset and as a function of the number of included single-cells.

Subsets of the enrichment dataset were assembled in a rarefaction analysis to determine the approximate genome size of *N. limnia* [figure 2]. The total assembly size smoothly asymptotes below 1.80 Mb, indicating that the *N. limnia* assembly represents an essentially complete genome at 1.77 Mb. We also rarefied the single-cell data, which we expected to assemble less efficiently than the enrichment dataset, due to the effect of greater amplification bias in the single cell MDA reactions compared with the bulk MDA of the enrichment sample. This expectation was borne out, but the single-cell data (in aggregate) do asymptote at approximately the same level. This suggests that the single-cell dataset contains all the genomic sequences, albeit with less genomic information per raw Mb as a result of representation bias. Assembly using only the single cell data yields a genome that is estimated to be 95% complete [Table 1]. It is interesting to imagine the impact of sequencing additional cells.

### Gene prediction and annotation

Gene prediction was carried out on the *N. limnia* assembly using the FgenesB platform (Softberry, Inc.), which called 2171 genes, including 2047 protein-coding ORFs, 74 pseudogenes, and 50 RNA genes [Table 2]. In total, 83.4% of the assembled bases were predicted to code for proteins. The *N. limnia* genome encodes 45 transfer RNA (tRNA) genes (42 identified by FgenesB and 3 additional tRNA sequences identified by comparison with *N. maritimus*). tRNAs for 18 amino acids were predicted, which further included two tRNAs with unidentified codon/amino acid specificity. Single copies of the full-length 16S, 23S, 5S, and 5.8S ribosomal RNA genes exist in the assembly. The average nucleotide identities of the 16S and 23S genes to *N. maritimus* are of 97% and 94%, respectively. In addition, a conserved noncoding RNA with 93% identity to *N. maritimus* was identified. The annotation data is reported in the supplementary information file Data S2.

**Table 2:** Genome features for *N. limnia*, *N. maritimus*, and *C. symbiosum* A.

Specifications	*N. limnia*	*N. maritimus*	*C. symbiosum* A
Size, bp	1,769,573	1,645,259	2,045,086
Average G+C content, %	32	34	58
Predicted genes	2,171	1,847	2,066
Average gene length (bp)	696	825	924
Coding percentage, %	83	90	91
**Gene content**			
Pseudogenes	74	6	0
Total number of ORFs	2,047	1,797	2,017
Predicted functional	1,136	1,082	994
Hypotheticals	911	714	1,023
ORF density (ORF/kb)	1.16	1.09	1.01
**RNA genes**			
16S-23S rRNA operon	1	1	1
5S rRNA	1	1	1
tRNAs	45	44	45
other RNA	2	2	1

### Codon usage and nucleotide skew

The *N. limnia* codon usage was compared with that of *N. maritimus* and *C. symbiosum* A, with a nucleotide-matched scrambled control as a reference (data not shown). The codon usage of *N. limnia* is very similar to that of *N. maritimus*, which has a similar G+C content, and distinct from both the high G+C content *C. symbiosum* A and the randomized control. This is also the case for *N. limnia* CDS lacking homology to database sequences, supporting the identification of these regions as protein-coding genes.

**Figure 3:**
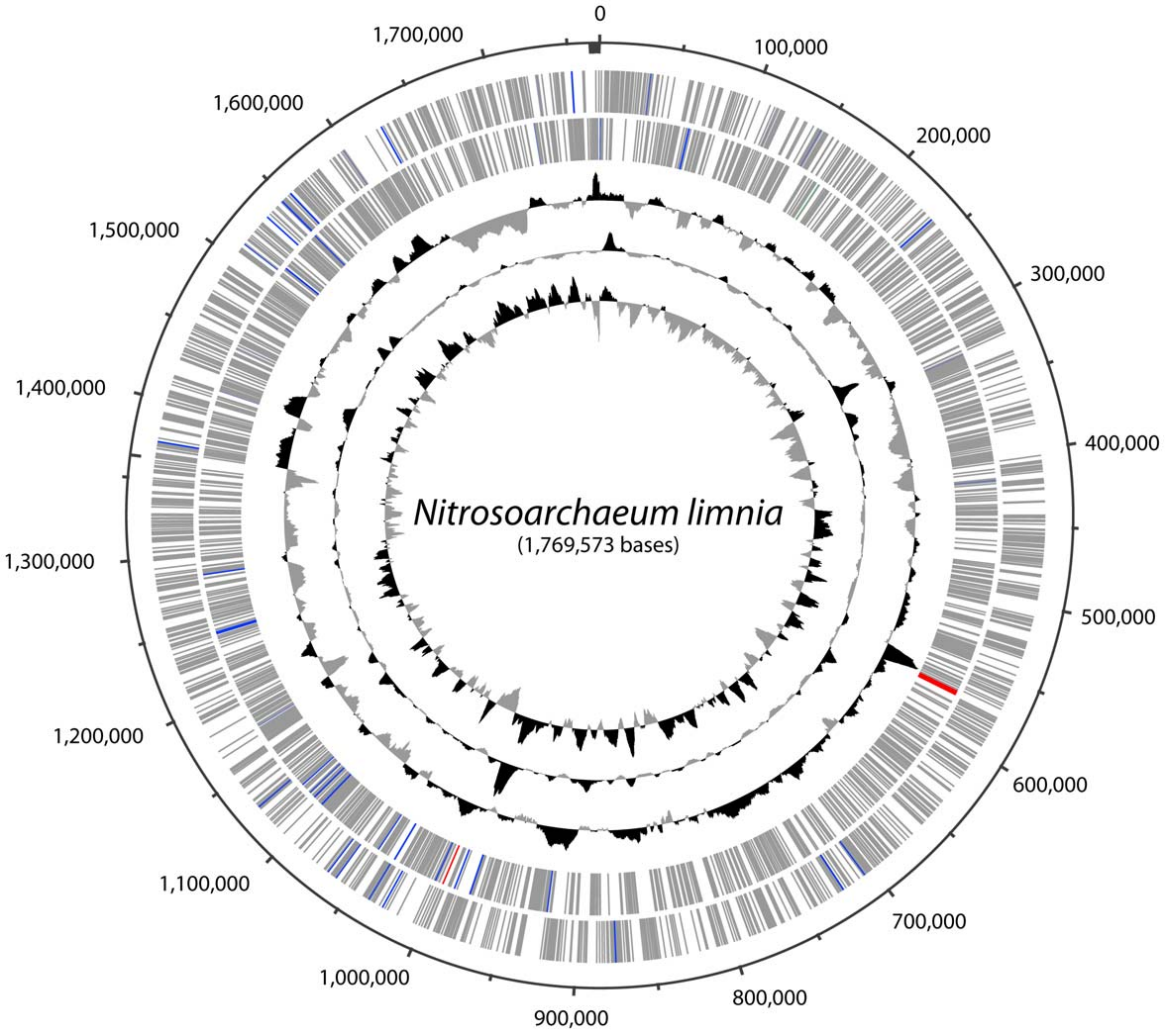
Circular representation of the *N. limnia* genome. From the outer ring to the inner ring: Scaffold breakpoints are indicated by the inside tick marks, predicted genes on the forward strand, predicted genes the reverse strand, G+C content, GC skew, and GGGT skew. On the outer two rings, protein coding sequences are colored gray, tRNA genes in blue, rRNA genes in red, and noncoding RNA genes in green.

The G+C skew in the *N. limnia* genome exhibits an irregular pattern comparable to that observed in other archaeal genomic sequences [figure 3] [37]. Analysis of cumulative G+C and GGG+T skew failed to reveal an obvious origin of replication, although several strong transition points exist in the genome.

### Gene prediction and percent identity comparisons

The *N. limnia* genome has a coding density of 1.16 protein coding genes per kb of sequence data, which is slightly higher than the coding density for *N. maritimus* (1.09) or *C. symbiosum* A (0.99) [Table 2]. The *N. limnia* genome shares 1423 genes with *N. maritimus* and 1156 genes with *C. symbiosum* A (based on Reciprocal Best Hit [RBH] analysis). The 1423 genes that were RBHs with *N. maritimus* genes represent 66% of the genes predicted in the *N. limnia* assembly. Conversely, 75% of the *N. maritimus* genes were identified as RBHs in the *N. limnia* assembly. Only 53% of genes in the *N. limnia* assembly were RBHs with *C. symbiosum* A, despite the larger genome size of *C. symbiosum* A, indicating the greater evolutionary distance that exists between *N. limnia* and *C. symbiosum* A than exists between *N. limnia* and *N. maritimus*.

**Figure 4:**
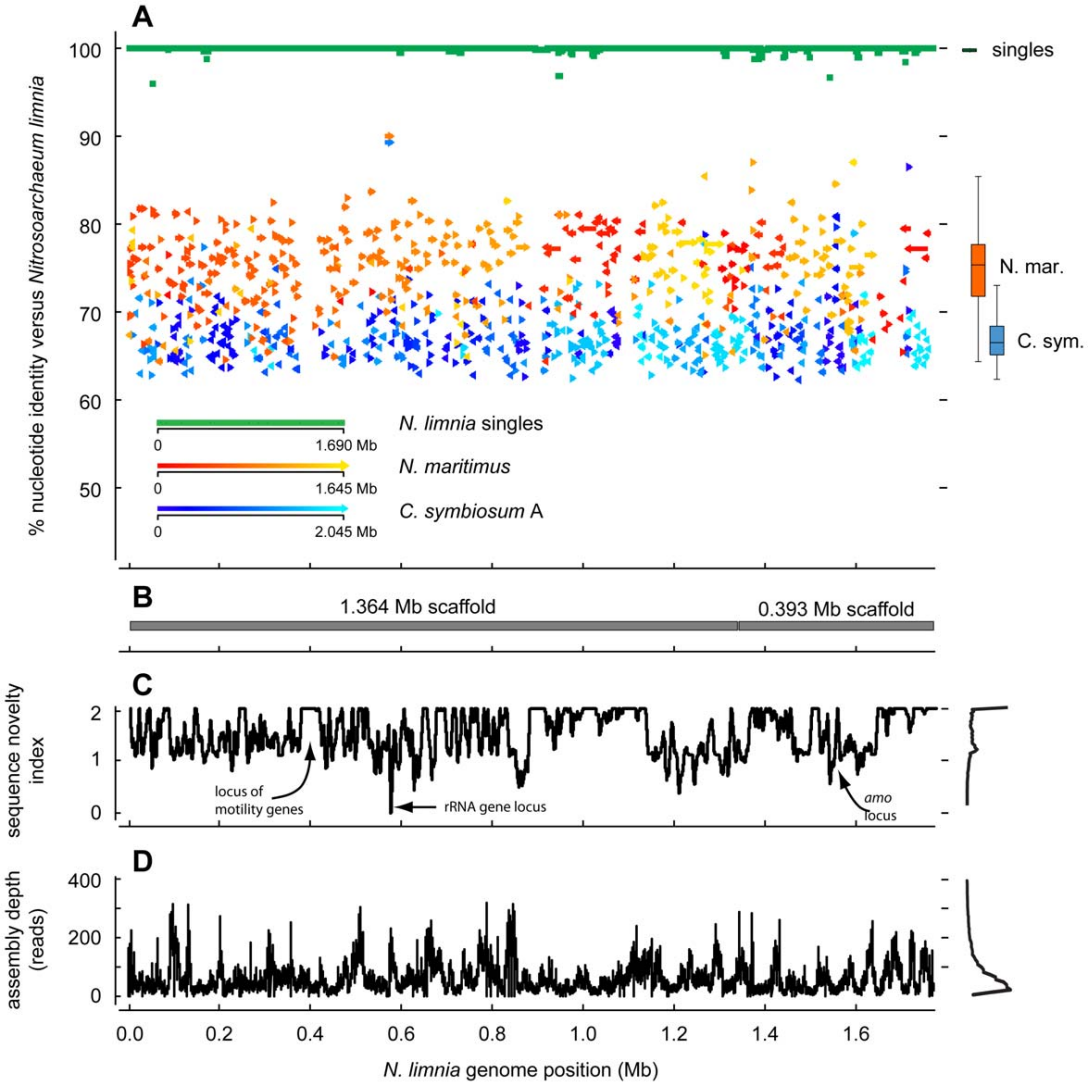
Comparison of three AOA genomes. (A) The top panel shows nucleotide identity of blast hits between the reference genomes and the *N. limnia* genome. Hits for *N. maritimus* and *C. symbiosum A* are colored according to the position in the reference (query) genome. The arrow direction indicates the hit direction. On the right, box plots summarize the distribution of hits from each reference genome to the *N. limnia* genome. (B) The positions of the two largest consensus scaffolds are shown. (C) The sequence novelty index, defined as 2 minus the sum of nucleotide identity of blast hits to *N. maritimus* and *C. symbiosum A* at each position is plotted. A histogram of the values is present at right. (D) The alignment depth in the final assembly is shown. A histogram of the values is present at right.

When compared across the entire length of the genome, *N. limnia* was distinct from both *N. maritimus* and *C. symbiosum* A [Table 3 and Figure 4]. The average nucleotide identity across overlapping regions of the *N. limnia* and *N. maritimus* genomes was 76.3% [Table 3]. When scoring non-overlapping genomic regions at an arbitrary 25% nucleotide identity, the overall nucleotide identity declines to 68.1%. The percent identity between the *N. limnia* and *C. symbiosum* A genomes was even lower, as expected due to the divergent total nucleotide content: 67.4% identity across overlapping regions and 33.3% when including non-overlapping regions [Table 3]. The cutoff for defining a new species was recently proposed to be 95–96% average nucleotide identity between two genomes [38]—an updated method of species identification compared to DNA-DNA hybridization. Thus, the much lower percent identity between *N. limnia*, *N. maritimus* and *C. symbiosum* A across the entire genome supports the classification of *N. limnia* as a new genus of AOA. The *N. limnia* and *N. maritimus* genomes shared extensive regions of synteny (gene order), whereas *N. limnia* and *C. symbiosum* A showed very little synteny across the genomes [Figure 4 and Figure S3].

**Table 3:** Whole genome nucleotide identity comparisons between *N. limnia*, *N. maritimus*, and *C. symbiosum A*.

Whole genome comparison	*N. maritimus*	*C. symbiosum* A
number of bases in corresponding regions	1,180,862	398,085
% identity of corresponding regions	76.3%	67.4%
whole genome average % identity*	68.1%	33.3%

*given 25% identity in non-corresponding regions

### Motility

One of the most striking features of the *N. limnia* genome, relative to *N. maritimus* and *C. symbiosum*, is the presence of numerous motility-associated genes, including those required for Flp pilus assembly, flagellar assembly, and chemotaxis [Table 4]. In total, *N. limnia* has 38 motility-associated genes, many of which are clustered in a locus on the largest scaffold [Figure 4]. This is a larger number than observed in many other archaea including *Sulfolobus solfataricus*, *Aeropyrum pernix*, and *Archaeoglobus fulgidus*, which all have fewer predicted motility genes (15–20 each). The *N. limnia* flagellar genes share homology with thermophilic Crenarchaeota. In addition, the majority of the cells in the AOA enrichment culture were observed to be motile by optical microscopy. This appears to be a distinctive trait from *N. maritimus* and *C. symbiosum* A, both of which lack gene suites for assembly of flagella. *N. limnia* was isolated from estuarine sediments, raising the possibility that this AOA requires motility to position itself in response to varying substrate and/or oxygen concentrations in the surface sediments. In contrast, *N. maritimus* was isolated from gravel in a tropical fish tank and *C. symbiosum* A is a symbiont of a marine sponge, where perhaps motility is less essential. Determining how widespread motility and chemotaxis are within phylogenetically diverse AOA genera (from water columns, soils, sediments, etc.) represents an exciting new avenue of research.

**Table 4:** *N. limnia* genes putatively associated with motility.

Motility Associated Genes	Number of Genes
**Flagella:**	
Archaeal flagella assembly protein J	1
Archaeal flagellins	5
Predicted ATPases involved in biogenesis of archaeal flagella	1
Type IV secretory pathway, VirB11 components, and related ATPases involved in archaeal flagella biosynthesis	1
**Chemotaxis:**	
Chemotaxis protein histidine kinase and related kinases	2
Chemotaxis protein; stimulates methylation of MCP proteins	1
Chemotaxis response regulator containing a CheY-like receiver domain and a methylesterase domain	2
Chemotaxis signal transduction protein	1
CheY-like receiver	19
Methyl-accepting chemotaxis protein	2
Methylase of chemotaxis methyl-accepting proteins	1
**Pilus:**	
Flp pilus assembly protein TadC	2

### Protection from osmotic shock

The *N. limnia* genome contains one large-conductance mechanosensitive channel protein and two small-conductance mechanosensitive channel proteins. Mechanosensitive (MS) channels are found in bacteria, archaea, and eukaryotes. MS channel proteins protect cells from hypoosmotic shock when a cell transitions from a high osmolarity environment to a low osmolarity environment [39]. This transition causes a rapid influx of water into the cells, which increases the internal turgor pressure and places considerable strain on the cytoplasmic membrane and cell wall. To release this pressure, the MS channels open, allowing the release of low molecular mass solutes (K^+^, glutamate and compatible solutes) out of the cell. This rapidly decreases the concentration of osmotically-active solutes in the cytoplasm, minimizing the influx of water and thereby preventing cell lysis. The *C. symbiosum* A and *N. maritimus* genomes appear to lack large-conductance MS channels, although they each encode at least one small-conductance MS channel protein. *N. limnia* was isolated from sediments in the San Francisco Bay estuary that likely experience regular fluctuations in salinity and may depend on these MS channel proteins for protection from osmotic shock.

The *N. maritimus* genome contains genes coding for ectoine biosynthesis, representing the first description of this process in Archaea [40]. Bacteria and Archaea accumulate many different organic osmolytes, including ectoine, in response to high osmotic pressure [41]. Notably, the *N. limnia* genome is missing all four genes for ectoine biosynthesis (ectA, ectB, ectC, and ectD). Although the *N. limnia* genome is not closed, the fact that the genome is missing all four genes suggests that *N. limnia* does not use ectoine biosynthesis as a mechanism for dealing with osmotic stress. These genomic differences may in turn reflect niche differentiation between *N. maritimus* (marine) and *N. limnia* (low-salinity).

### Genes conferring resistance

Several phage integrase proteins were identified in the *N. limnia* genome. However, no obvious CRISPRs (clustered regularly interspaced short palindromic repeats) were detected in the genome, which confer resistance to phage infection in many archaeal species, despite the fact that CRISPRs are found within ∼90% of archaeal genomes [42] and references within]. Thus, *N. limnia* may martial other defenses—the genome codes for at least one restriction-modification systems—or may be particularly susceptible to phage infection. Three transposase genes were also present in the genome and may be an additional source of genetic exchange and rearrangement. Several genes putatively conferring antibiotic resistance were found in the *N. limnia* genome (*e.g.* beta-lactamase domain-containing proteins and an antibiotic biosynthesis monooxygenase). The presence of several metal transporters and proteins implicated in metal resistance may reflect metal exposure in the San Francisco Bay sediments; indeed, there are many potentially toxic metals in the sediments of North San Francisco Bay [23].

### Novel cell division system

In Euryarchaeota and Korarchaeota (as well as most bacteria), cell division is mediated by FtsZ proteins, which are homologous to tubulin, the building block of the microtubule cytoskeleton in eukaryotes [43]–[44]. The sequenced genomes of hyperthermophilic Crenarchaeota lack FtsZ genes. Instead, it appears that all Crenarchaeota (with the exception of the Thermoproteales, where the cell division machinery remains uncharacterized) use a novel Cdv cell division system that is related to the related to the eukaryotic ESCRT-III sorting complex [44]–[45]. However, *both* the *FtsZ* and *Cdv* systems of cell division are evident in the AOA genomes, including *N. limnia*, *N. maritimus*, and *C. symbiosum* A, as well as the draft genome of *Nitrososphaera gargensis* [20]. The striking difference in cell division between these AOA and all other Crenarchaea supports the proposal that they belong to a third archaeal phylum, the Thaumarchaeota [19]–[20].

### Chromosome structure and organization

Prokaryotic chromosomal DNA is highly compacted to form a nucleus-like structure—the nucleoid. Bacteria and Archaea have to compact their genomic DNA by over 1000-fold [46] by utilizing a wide variety of DNA binding chromatin proteins. These proteins not only serve to compact the genomic DNA, but also in maintaining the genome in a state that allows access for gene expression, DNA replication and repair. The two major archaeal chromatin proteins are the archaeal histone and Alba proteins. Archaeal histones act as spools that DNA wraps around (similar to their eukaryotic homologs), whereas Alba proteins form extended filamentous fibers containing DNA [47]. All archaea have either one of these proteins, and many encode both proteins [48]. The *N. limnia* genome codes for one histone protein (Nlim1809) and two Alba proteins (Nlim0610 and Nlim1213).

### Carbon metabolism

The *N. limnia* genome sequence suggests that this organism uses a modified version of the 3-hydroxypropionate/4-hydroxybutyrate pathway for carbon fixation, as in *N. maritimus* and *C. symbiosum* A [37], [52]. In addition to pathways for autotrophic carbon fixation, genes putatively inferring the function of organic carbon consumption have been identified in the genomes of *N. limnia*, *N. maritimus* and *C. symbiosum* A. Several isotopic studies have suggested that natural populations of AOA may be capable of mixotrophic growth [4]–[5], [49]–[51]. It remains to be determined how important heterotrophy and/or mixotrophy is to AOA in natural environments.

### Ammonia oxidation and respiratory chain

In ammonia-oxidizing bacteria (AOB), ammonia is oxidized to hydroxylamine by ammonia monooxygenase (AMO) composed of α, β, and γ subunits encoded by the *amoA*, *amoB*, and *amoC* genes, respectively. Hydroxylamine oxidoreductase (HAO) then oxidizes hydroxylamine to nitrite, generating four electrons that are transferred to the terminal oxidase and AMO via cytochromes *c*554 and *c*_M_552 and the ubiquinone pool ([52], [53] and references therein).

The *N. limnia* genome contains genes putatively encoding the α, β, and γ subunits of the archaeal AMO protein. The gene order in the AMO operon is conserved amongst *N. limnia*, *N. maritimus* [40], and the Sargasso Sea metagenomic fragments [54]: *amoA*, hypothetical protein, *amoC*, followed by *amoB*. The *N. limnia* AMO gene sequences have relatively low identities to *N. maritimus*, ranging from 80–91% nucleotide identity and 77–96% amino acid identity [Table 5].

**Table 5:** Nucleotide (NT) and amino acid (AA) identities for key individual genes in *N. limnia*, *N. maritimus*, and *C. symbiosum* A.

	*N. limnia* vs		*N. limnia* vs	
	*N. maritimus*		*C. symbiosum* A	
Individual gene comparison	*AA Identity*	*NT Identity*	*AA Identity*	*NT Identity*
**16 s rRNA**	n/a	97%	n/a	94%
**23 s rRNA**	n/a	94%	n/a	89%
**ammonia monooxygenase subunit A**	95%	89%	95%	78%
**ammonia monooxygenase subunit B**	92%	88%	76%	75%
**ammonia monooxygenase subunit C**	96%	91%	93%	81%
**AMO-associated hypothetical protein** **	77%	80%	73%	72%***

**ammonia monooxygenase operon-associated hypothetical protein

***<90% sequence coverage

While the details of the ammonia oxidation and electron transfer pathways remain to be elucidated, AOA, including *N. maritimus*, *C. symbiosum* A and *N. limnia*, appear to have an ammonia oxidation pathway distinct from AOB [40],[55]–[56]. The *N. maritimus*, *C. symbiosum* A and *N. limnia* genomes lack homologues for HAO or cytochromes *c*554 and *c*_M_552, and it remains to be demonstrated whether hydroxylamine is an intermediate in the pathway. AOA in pure culture produce nitrite from the oxidation of ammonia [21], [57] but the genome sequence annotations do not include the key HAO enzyme necessary to produce nitrite. The archaeal AMO protein may not produce hydroxylamine (explaining the missing HAO protein) or the AOA may contain novel enzymes responsible for the oxidation of hydroxylamine. In either case, redox reactions in AOA are likely more reliant on copper than iron as seen in the AOB with the iron-rich HAO enzyme and cytochromes [40], [55]. The three AOA genomes all contain numerous copper-containing proteins, including multicopper oxidases (MCOs) and small blue copper-containing proteins. These predicted copper-containing proteins might form the basis of electron transfer in AOA with functionality similar to cytochromes in AOB [40], [55]. The *N. limnia* genome contains 11 blue (type 1) copper domain-containing proteins, 1 cupredoxin blue copper protein, and 4 multicopper oxidases [Table S1]. Alternative pathways for ammonia oxidation have been proposed by Walker et al. [40]. The *N. limnia* genome does not further illuminate the exact mechanism for ammonia oxidation.

Two ammonium transporter genes were identified in the *N. limnia* genome (Nlim1421 and Nlim1564). If the AMO protein accesses ammonia outside of the cytoplasmic membrane (as in AOB), the AOA would not necessarily need ammonium transporters. However, the RBHs exist between the *N. limnia* ammonium permeases and both *N. maritimus* and *C. symbiosum* A. The existence of conserved ammonium transporters in all three genomes suggests a necessary function for this activity. Arp et al. (2007) [52] proposed that ammonium transporters in AOB might facilitate the regulation of AMO and HAO that is induced by ammonium or to assist in the uptake of ammonia for nitrogen assimilation.

Many AOB are able to use urea as an alternative source of ammonia and carbon dioxide for growth [58]. Ureolysis may therefore be an ecological advantage in environments with low pH and/or fluctuating ammonia and carbon dioxide availability. Like *N. maritimus* [40], there was no evidence that *N. limnia* has the ability to use urea as an alternative source of ammonia, because no genes encoding urea transporters or urease enzymes were identified in the genome. Interestingly, however, both urea transporter and urease genes were present in the *C. symbiosum* A genome [55]. The symbiotic *C. symbiosum* A may use ureolysis as a mechanism to remove nitrogenous waste from its host, the marine sponge *Axinella mexicana* [56]. Konstantinidis et al. [16] also reported urease genes in a crenarchaeal genomic scaffold from 4,000-m-depth at station ALOHA in the Pacific Ocean (along with *amoA*, *amoB*, *amoC*, and ammonia permease genes), suggesting that urease activity is a feature of at least some marine water column AOA.

### Conclusion

We used an approach combining metagenomic and single cell data to produce a high quality and complete (but not fully closed) genome of a mesophilic AOA from a low-salinity estuarine environment – *Nitrosoarchaeum limnia*. Single-cell genome sequencing enabled discovery and assembly of 95% of the complete *N. limnia* genome; when this data was combined with metagenomic reads derived from the bulk enrichment culture, greater than 97% of the genome was determined. The genomic sequence reflects several systems present in the two other AOA genome sequences available, including the machinery for cell division and ammonia oxidation, establishing common elements that define this functionally related group of archaea belonging to the proposed archaeal phylum Thaumarchaeota. The *N. limnia* genome also reveals features unique to this environmental AOA, including a substantial suite of genes for flagellar biosynthesis and chemotaxis, and the presence of a large mechanosensitive channel that may be crucial for fitness in the estuarine environment. Several large regions of the genome lacking homology to *N. maritimus* and *C. symbiosum* may encode other functional genes important to the survival of free-living, estuarine AOA. Cultivation and genomic sequencing of environmentally-relevant AOA portends further physiological and metabolic studies of this novel and pervasive group of organisms that will ultimately advance our understanding of the global carbon and nitrogen cycles.

## Materials and Methods

### Cultivation of archaeal ammonia-oxidizers

Ammonia-oxidizer enrichment cultures were initiated from sediments collected in northern part of the San Francisco Bay estuary in 2006 (site SU001S; latitude 38°5′55.75″N, longitude 122° 2′47.40″W). Surface sediments were inoculated into a modified low-salinity version of Synthetic Crenarchaeota Medium [21], as described by Mosier and Francis (2008) [23]. Cultures were grown in static culture flasks or serum vials in the dark at room temperature (approximately 22°C). Approximately 10% of the culture volume was routinely transferred into fresh media.

The presence of ammonia-oxidizing archaea was confirmed by gene sequencing and fluorescent in situ hybridization (FISH). Briefly, DNA was extracted from the enrichment culture and archaeal 16S rRNA (Arch21F and Arch958R primers; [1]) and archaeal *amoA* (Arch-amoAF and Arch-amoAR primers; [59]) genes were PCR amplified, cloned and sequenced. Catalyzed reporter deposition fluorescence in situ hybridization (CARD-FISH; e.g., [60]) was carried out with horseradish peroxidase (HRP)-labeled probes Arch915 [61] and EUB338 I–III [62]–[63]. Filters were treated with lysozyme or proteinase K for permeabilization of bacteria and archaea, respectively.

### Phylogenetic trees

Archaeal *amoA* nucleotide sequences were aligned with GenBank sequences using MacClade version 4.08 [64]. Archaeal 16S rRNA gene sequences were aligned using the NAST alignment server [65]. Sequences were then manually adjusted and aligned to the Greengenes 16S rRNA gene database [66] in ARB [67]. For phylogenetic analyses, a 582-bp region of the sequence alignment was used for *amoA* and a 1272-bp region for 16S rRNA. Neighbor joining (based on Jukes-Cantor correction) and parsimony phylogenetic trees were constructed in ARB [67] with 1000 bootstrap replicates. The set root option in ARB was used to manually root the trees between the sediment and water column cluster sequences.

### Microfluidic device and cell sorting

Microfludidic devices with 32 units for nanoliter MDA reactions were produced by the Stanford microfluidics foundry. These devices are similar to those described in Marcy *et al.* [26]. The chip was pre-treated for 10 minutes with pluronic F127 at 0.2% in 1× PBS before filling with 1x PBS containing 0.01% Tween-20 and 0.01% pluronic F127 to reduce cell adhesion. The sample was pre-treated with 0.5–1 µg/mL proteinase K at room temperature for 10 minutes and then washed in 1× PBS and resuspended in 50∶50 1× PBS∶Ethanol. BSA was added to the treated cells (0.1 mg/mL final concentration). Individual cells were sorted one at a time from the bulk sample using the laser trap, through a two-valve gate, opening one valve at a time to allow the trapped cell, but not fluid flow, to pass through (see Movie S1). Each trapped cell was moved about 1 mm from the bulk sample to the reaction chamber using the laser trap.

### Single-cell amplification

The Repli-G midi MDA reagents (Qiagen) were used to amplify individual cells in 50–60 nL volumes on the device, yielding 10^7^–10^8^ genomic equivelants of dsDNA product. First, cells contained in 0.75 nL PBS with 0.02% Tween-20 were flushed into the first lysis chamber with 3 nL buffer DLB (supplemented with 0.1 M dithiothreitol) to complete lysis and denature the genomic DNA. Then, ∼50 nL of reaction mix (45 µL were prepared from 29 µL Repli-G reaction buffer, 10 µL 20 mM H_2_O with 0.6% tween, 2 µL Repli-G enzyme, and 2 µL Repli-G stop solution) was added to each of the 32 reactions. The chip was then transferred to a hot plate set to 32°C and incubated overnight. This was carried out by fitting the outboard product ports on the chip with plastic pipet tips (P10 size), and by flushing the products into the pipet tips with the TRIS solution plumbed into the reagent port at 8 psi.

Six µl of each first-round reaction product were re-amplified using the Repli-G midi kit (Qiagen). Additionally, 1 µl of the proteinase K-treated cell solution used for sorting was amplified. The template solution was denatured by the addition of 3.5 µl buffer DLB for 5 minutes at room temperature. The solution was neutralized by adddition of 3.5 µl stop solution. A reaction mix consisting of 29 µl reaction buffer, 10 µl water, and 1 µl enzyme was prepared on ice, and then added to the denatured template. Reactions were incubated at 30°C for 12 hours and then diluted 10-fold in 10 mM TRIS with 0.02% Tween-20 and stored at −60°C.

### Shotgun and paired-end library prep

Shotgun libraries were prepared from approximately 5 µg of the second round MDA product according to the Roche/454 protocol for “Titanium” shotgun libraries with the following modifications. Custom barcoded adaptor oligos (IDT) were used to enable pooling multiple libaries in a single emulsion PCR reaction and picotiter plate region during sequencing. To obtain dsDNA sequencing libraries and shorten the library preparation process, the library immobilization, Fill-in, and single-stranded library isolation steps (steps 3.7–3.9) were omitted.

Paired end libraries were prepared from approximately 5 µg of the second round MDA product according to the Roche/454 protocol for “Titanium 3 kb span” libraries with the following modifications. Custom barcoded adaptor oligos (IDT) were used in step 3.9.2 to enable pooling multiple libaries in a single emulsion PCR reaction and picotiter plate region during sequencing. To obtain dsDNA sequencing libraries and shorten the library preparation process, the library immobilization and single-stranded library isolation steps (steps 3.12.1 and 3.12.2) were omitted.

### Sequencing library quantification

Sequencing libraries were quantified using digital PCR as previously reported [36], with the exceptions that 48.770 digital arrays (Fluidigm) were used for the microfluidic dPCR step, and that amplification primers complimentary to the Titanium adaptor sequences were used. Briefly, serial dilutions of the sequencing libraries were made in 10 mM TRIS buffer with 0.02% Tween-20. 48 sample preparations were then combined per the Fluidigm dPCR protocol with a reaction buffer containing thermostable DNA polymerase, dNTPs, GE sample loading reagent (Fluidigm), and the primers and probe necessary to carry out the universal Taqman amplification/detection scheme. The samples were loaded in the chip and run on the Biomark thermocycler for 45 cycles. Sample analysis was carried out using the default parameters for dPCR analysis using the Fluidigm analysis software. The quantitated libaries were then diluted to 2×10^6^ molecules per microliter in 10 mM TRIS with 0.02% Tween-20 and aliquotted for storage at −60°C.

### Genome sequencing

DNA pyrosequencing of the shotgun and paired-end libraries was carried out on the Roche 454 Genome Sequencer FLX instrument using “Titanium” chemistry. Emulsion PCR was carried out with DNA∶Bead ratios of 0.3∶1 for the shotgun libraries and 2.5∶1 for the paired-end libraries.

### Sequence assembly

Reads from the shotgun and paired end pyrosequencing runs were separated by sample and trimmed using the sfffile tool (Roche) permitting one error in each 10 bp barcode. The G+C content of the single-cell reads formed a major peak centered at 32.4%, about 2% lower than the average G+C content of *N. maritimus* (34.2%). The dispersion of the read G+C content was essentially identical to that of *N. maritimus*, with the latter sampled at the same average read length. The few reads with G+C content greater than 46% were mapped to the *N. maritimus* genome, and mapping reads (90% identity over 40 bases) were combined with the low G+C content reads for subsequent analyses. The reads from the enrichment sample were then assembled de novo using Newbler (Roche). All assembly operations were carried out with Newbler v2.3 using default parameters. Paired end reads from the enrichment sample tagged by the assembler as ‘chimeric’ were excluded from subsequent operations. Reads from the single cells were then mapped to the initial enrichment assembly, and ‘chimeric’ reads excluded. Contigs made up of single-cell reads were then sampled as 550-mers with 50 base overlaps and included as reads in the final de novo assembly of the single-cell data. A second, ‘consensus’, de novo assembly was also perfomed using the single cell pyrosequencing reads (∼120 Mb) and the reads from the enrichment (∼37 Mb). A small number of short outlier contigs were excluded from the final assembly due to the identification of sequences from known contaminants from the MDA kit, such as *Delftia acidovorans*.

### Gene annotation

Genes were predicted on the assembled genome sequence using the FgenesB program (Softberry, Inc.). Automated FgenesB annotations were manually refined by reviewing Reciprocal Best Hits (RBH) to *Nitrosopumilus maritimus* and *Cenarchaeum symbiosum* A and BLAST searches against the GenBank database. Individual proteins of interested were evaluated against “InterPro: the integrative protein signature database” [68] using InterProScan. RBH were identified using the blastp algorithm of Blast+ in conjunction with custom Matlab code. RBH are classified as “high confidence” when the forward hit covered at least 60% of the query sequence for the gene pair. Pseudogenes lacking a start or stop codon (74 in total) were included in the gene-based analyses. In total, 1200 genes were functionally annotated and 607 have strong homology to another organism but no assigned function. 323 unannotated genes (156 of which are 30 aa in length or less) were labeled as “hypothetical proteins.”

### Genome sequence analysis

CRISPRFinder [69] and the CRISPR recognition tool (CRT) [70] was used to search for Clustered Regularly Interspaced Short Palindromic Repeats (CRISPRs) using the default settings. Synteny plots were created using the Nano+Bio-Center computational biology software (http://nbc3.biologie.uni-kl.de/). Comparisons were made at the DNA level using Nucmer and then the protein level using Promer by translating the DNA sequences in all six reading frames. Analyses of nucleotide content and sequence read statistics were performed using Blast+ on a 64 bit Linux server and using custom Matlab and Perl code.

## Supporting Information

Figure S1 Phase contrast images of the five single cells subjected to single-cell whole genome amplification and genome sequencing. The small cells are indicated by circles; scale bar corresponds to 1 micrometer.
(TIF)

Figure S2 GC content of raw 454 reads from the 5 single cells and the AOA enrichment. The GC content of the filtered set of reads used for the final assembly of *N. limnia* and simulated reads from the *N. maritimus* genome are also shown.
(TIF)

Figure S3 Synteny plots relating N. limnia to the N. maritimus and C. symbiosum A genomes.
(TIF)

Table S1 Genes in *N. limnia* that are putatively involved in ammonia oxidation and respiration (Table modified from Walker *et al.*, 2010).
(XLS)

Movie S1 Movie shows last stage of sorting cell 90507.21 (occurring in the area on the device beyond the second gate valve). The laser trap is fixed near the end of the fiducial arrow. In the movie, the cell is moved some 10 s of micron before the stage is stopped. The trap beam is interrupted when the stage is stopped, freeing the cell to diffuse away. The pixels visible in the fiducial arrow are 0.106 micron wide.
(AVI)

Data S1 The genome sequence is provided as a fasta file.
(TXT)

Data S2 A spreadsheet containing the annotation data is provided. At the time of writing, this and the genome sequence data are in submission at the NCBI.
(XLSX)
