# Supplementary figures and images for: Genome of a Low-Salinity Ammonia-Oxidizing Archaeon Determined by Single-Cell and Metagenomic Analysis
Source: PLoS One. 2011 Feb 22;6(2):e16626. doi: 10.1371/journal.pone.0016626 (PMC3043068; doi:10.1371/journal.pone.0016626)

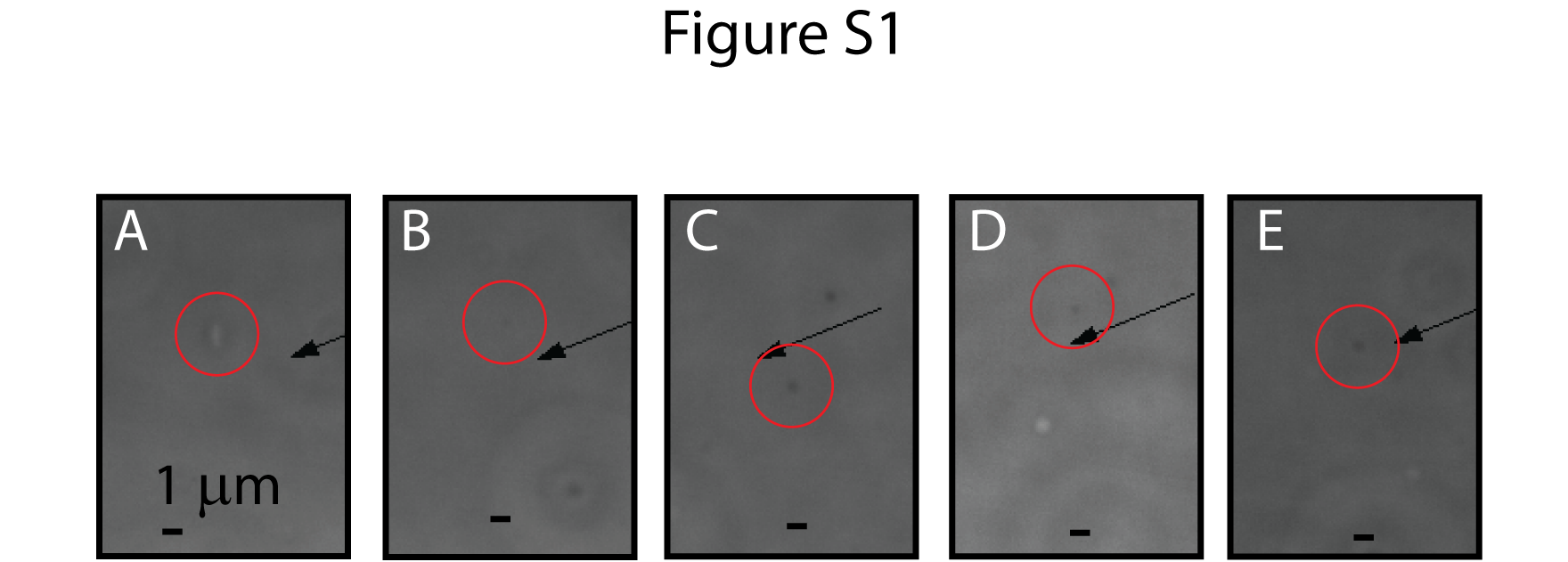

Supplement: Figure S1 — Phase contrast images of the five single cells subjected to single-cell whole genome amplification and genome sequencing. The small cells are indicated by circles; scale bar corresponds to 1 micrometer. (TIF) [file pone.0016626.s001.tif]

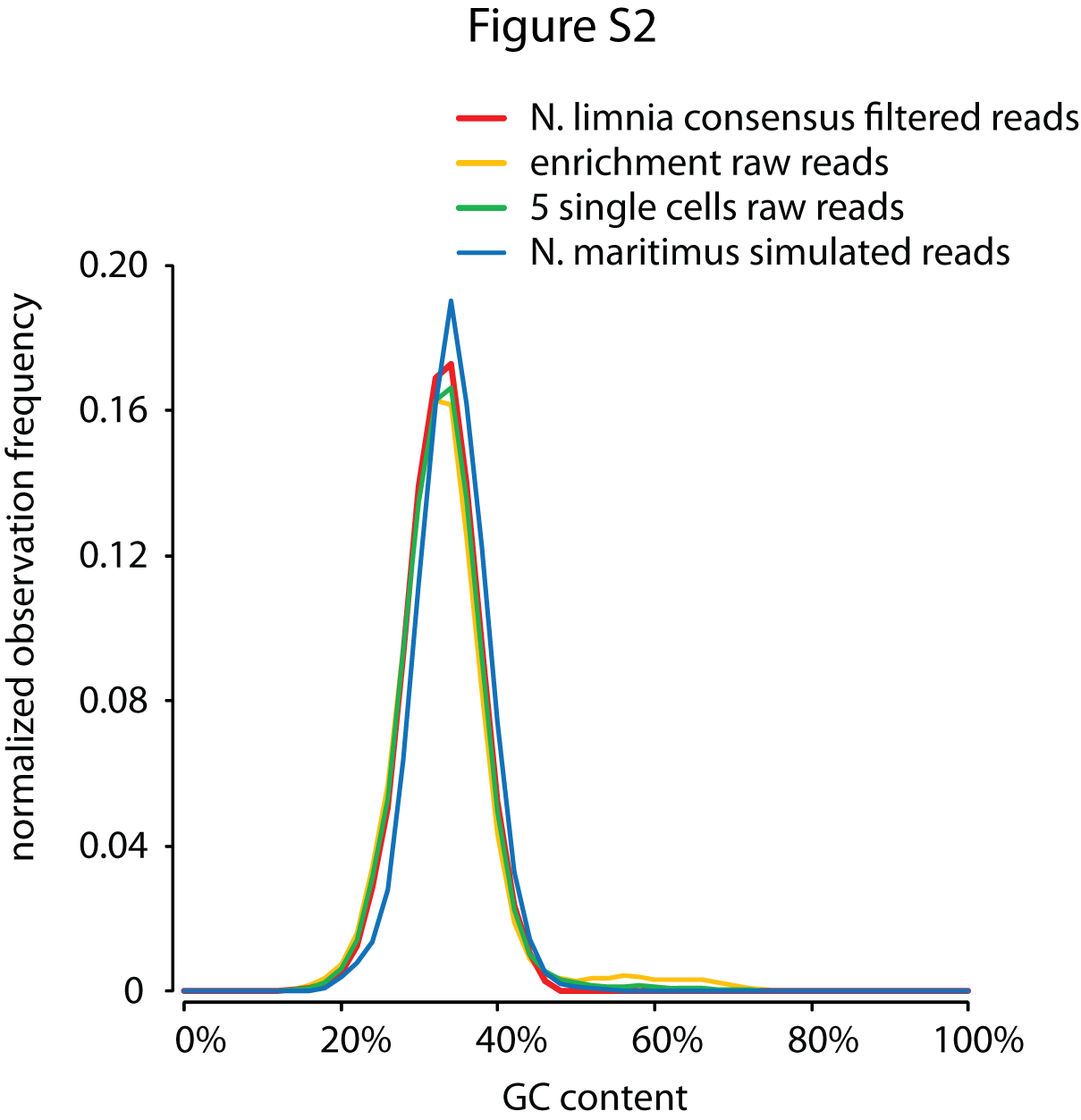

Supplement: Figure S2 — GC content of raw 454 reads from the 5 single cells and the AOA enrichment. The GC content of the filtered set of reads used for the final assembly of N. limnia and simulated reads from the N. maritimus genome are also shown. (TIF) [file pone.0016626.s002.tif]

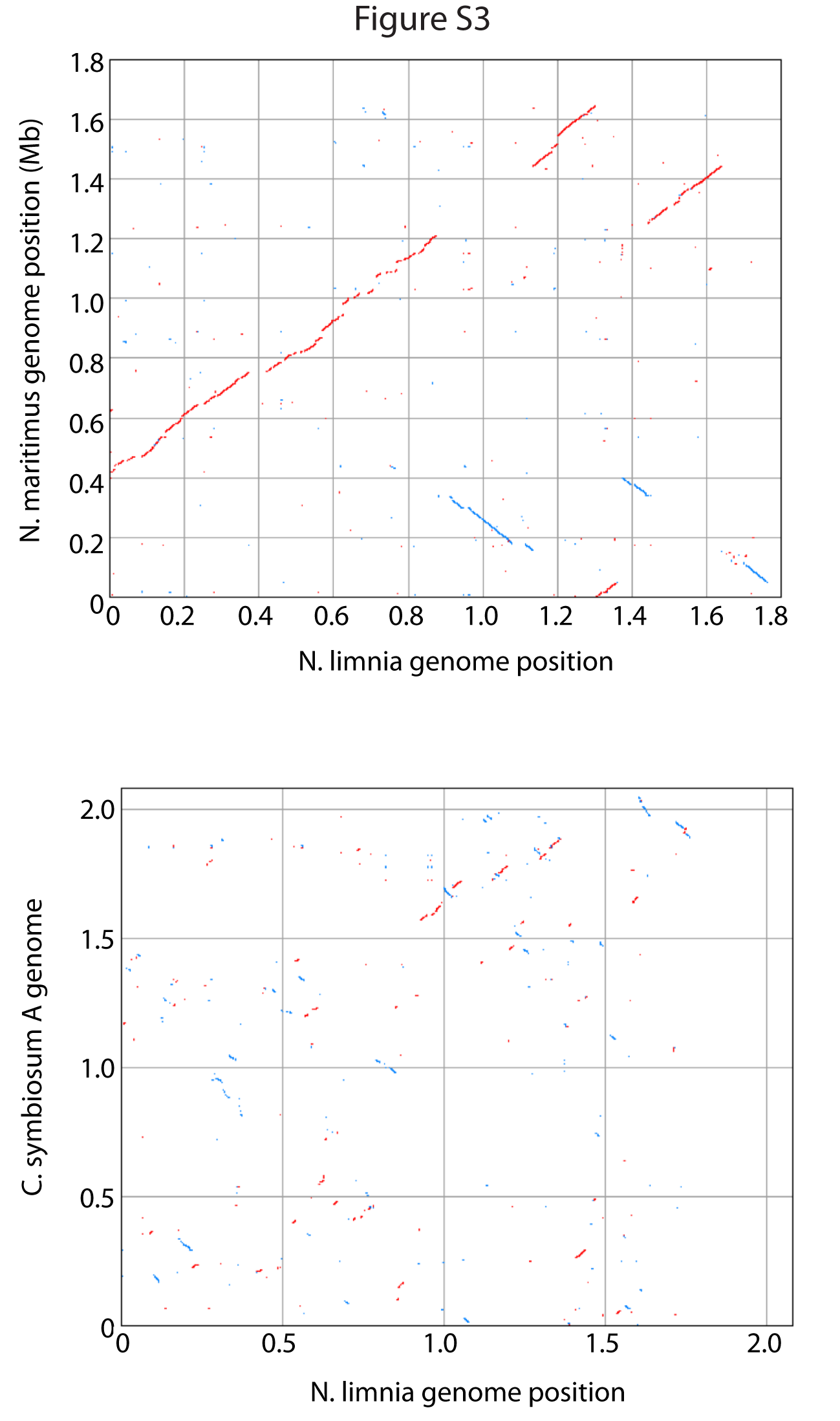

Supplement: Figure S3 — Synteny plots relating N. limnia to the N. maritimus and C. symbiosum A genomes. (TIF) [file pone.0016626.s003.tif]
